# Renal Function Is Associated with Changes in Bone Mineral Density in Postmenopausal Osteoporotic Women Treated with Denosumab: Data From a Retrospective Cohort Study

**DOI:** 10.3390/jcm13206239

**Published:** 2024-10-19

**Authors:** Antonino Catalano, Cecilia Oliveri, Giuseppe Natale, Rita Maria Agostino, Giovanni Squadrito, Agostino Gaudio, Guido Gembillo, Djordje Marina, Valeria Cernaro, Elisa Longhitano, Giorgio Basile, Nunziata Morabito, Domenico Santoro

**Affiliations:** 1 Unit and School of Geriatrics, Department of Clinical and Experimental Medicine, University of Messina, Policlinico “G. Martino”, 98124 Messina, Italy; 2 Mineral Metabolism and Nephrology Clinic of Vibo Valentia Hospital, 89900 Vibo Valentia, Italy; 3 Unit of Oncology, Grand Metropolitan Hospital “Bianchi Melacrino Morelli”, 89133 Reggio Calabria, Italy; 4 Unit and School of Internal Medicine, Department of Clinical and Experimental Medicine, University of Messina, 98124 Messina, Italy; 5 Unit and School of Internal Medicine, Department of Clinical and Experimental Medicine, University of Catania, Policlinico “G. Rodolico”, 95123 Catania, Italy; agostino.gaudio@gmail.com; 6 Unit and School of Nephrology, Policlinico “G. Martino”, Department of Clinical and Experimental Medicine, University of Messina, 98124 Messina, Italy; 7 Department of Nephrology and Endocrinology, Rigshospitalet, University of Copenhagen, 1017 Copenhagen, Denmark; dorde.marina.02@regionh.dk

**Keywords:** renal function, denosumab, osteoporosis, bone mineral density, postmenopause, glomerular filtration rate

## Abstract

**Background/Objectives**: Renal function influences bone metabolism, as kidney failure can increase the risk of fractures. Denosumab is an approved osteoporosis treatment, but its efficacy in relation to renal function has not yet been studied in real-life scenarios. This study aimed to investigate the denosumab-induced change in bone mineral density (BMD) according to kidney function. **Methods**: A retrospective analysis was conducted at the outpatient clinic in postmenopausal women receiving denosumab (60 mg subcutaneously administered every 6 months). The glomerular filtration rate (eGFR) was measured by the 2021 CKD-EPI equation and patients were stratified for eGFR categories. BMD was measured by dual-energy X-ray absorptiometry. **Results**: 128 women (mean age 70.3 ± 9.4 years) were recruited. The mean denosumab treatment duration was 3.9 ± 1.4 years and all the participants had improved BMD values. In stepwise multiple regression analysis—after controlling for age, BMI, and treatment duration—the eGFR value (ß = −0.11, SE 0.04, *p* = 0.01) was independently associated with the lumbar spine BMD change. The same association remained when the eGFR categories were considered (ß = 3.564, SE 1.29, *p* = 0.007). In addition, after controlling for BMI and the duration of denosumab treatment, age (ß = −0.7915, SE 0.37, *p* = 0.03) and eGFR (ß = −0.3257, SE 0.1567, *p* = 0.04) were found to be associated with femoral neck BMD change. The association remained when considering eGFR categories (ß = 8.7339, SE 4.29, *p* = 0.04). **Conclusions**: This retrospective study suggests that eGFR is associated with denosumab efficacy in postmenopausal women treated for osteoporosis.

## 1. Introduction

Osteoporosis is increasingly recognized as a systemic disease. It is primarily observed in women and the elderly, leading to increased susceptibility to fracture and disability and imposing a significant socioeconomic burden. Fragility fractures, occurring due to osteoporosis, are also associated with a higher mortality rate [1].

Osteoporosis is estimated to affect 200 million women worldwide, with prevalence increasing with age. It affects one in ten women at the age of 60, one in five at the age of 70, two in five at the age of 80, and two in three at the age of 90 [2].

As the world population ages—based on the projected number of fractures calculated using age- and sex-specific incidence, and population size at 5-year intervals from 2019 to 2034 [3]—it is expected that the annual number of osteoporotic fractures in the EU27 + 2 will increase by 1.06 million, from 4.28 million in 2019 to 5.34 million in 2034 [4]. The declining incidence of hip fractures observed in many countries in recent years is unlikely to offset the impact of the aging population. As a result, the number of hip fractures is anticipated to almost double over the next 20 to 30 years [5].

Advances in the pharmacologic treatment of osteoporosis are essential for reducing the incidence and improving the outcomes of bone fragility fractures. Ongoing research and targeted medical interventions are critical for the effective treatment of osteoporosis, particularly in aging populations and patients with comorbidities. Several drugs have been approved for the medical treatment of osteoporosis, including antiresorptive drugs (e.g., bisphosphonates (BPs) and denosumab (Dmab)), anabolic treatments (e.g., teriparatide, abaloparatide) and dual-acting bone builder agents (e.g., romosozumab). [6]

BPs—which include alendronate, risedronate, ibandronate and zoledronic acid—are the most used antiresorptive drugs in the treatment of osteoporosis due to their efficacy in reducing the risk of fractures [7]. The pharmacokinetics of BPs are associated with significant renal excretion. After administration, BPs bind to the bone mineral surfaces and the excess drug that is not captured by the bones is excreted via the kidneys [8].

According to the Food and Drugs Administration (FDA) recommendations, the use of BPs is contraindicated in patients with severe renal impairment, defined as a glomerular filtration rate (eGFR) less than 30 mL/min, due to the potential risk of nephrotoxicity, including acute kidney injury. [9]

Dmab, a monoclonal antibody that inhibits the nuclear factor-kappa B receptor-activating ligand (RANKL), is a potent antiresorptive agent [10]. The FREEDOM study showed that Dmab significantly reduced the risk of fragility fractures in postmenopausal women [11]. According to the FDA, the efficacy and safety of Dmab vary depending on the patient population considered. The benefit of Dmab is evidenced by the reduction in vertebral, non-vertebral, and hip fractures, making it a versatile option in the management of osteoporosis [11]. The FREEDOM study also showed Dmab was effective and consistently safe in different stages of renal disease, even in patients with chronic kidney disease (CKD). Further studies also suggested that it was safe to administer Dmab to patients with advanced CKD, and to those on hemodialysis [12,13,14]. Furthermore, a meta-analysis investigating osteoporosis medications highlighted bone mineral density (BMD) improvement with Dmab in kidney transplant recipients and patients with CKD [15].

To our knowledge, the impact of renal function on the Dmab efficacy has not been studied in a real-life setting. Therefore, the main aim of this study was to explore BMD changes due to Dmab treatment and its association with renal function in osteoporotic postmenopausal women at high fracture risk.

## 2. Materials and Methods

This study’s participants were postmenopausal women at high risk of fracture who were referred to the Outpatient Clinic for the Prevention and Treatment of Metabolic Bone Disease, at the Department of Clinical and Experimental Medicine, University Hospital of Messina, Italy, between 2018 and 2023. Patients with a current or previous history of malignancies, liver failure, heart failure, CKD stage G5 or dialysis, hyperthyroidism, hypoparathyroidism or hyperparathyroidism, hypercalcemia or hypocalcemia were not included. Patients were recruited if they had received treatment with Dmab for the secondary prevention of fragility fractures at the time of recruitment, as per the Italian drug reimbursement regulations. They had at least one prevalent morphometric vertebral fracture, defined according to the Genant criteria based on vertebral shape in terms of loss of vertebral height loss in the anterior, posterior, and/or middle vertebral body. The availability of BMD measurements—at least one preceding Dmab treatment and within the last six months before study entry, and one at the end of the study—was a prerequisite for patients’ eligibility. Patients were included in this study if they maintained treatment with Dmab for at least 18 months, without discontinuing it. This study was conducted according to the guidelines of the Declaration of Helsinki and approved by the Ethics Committee at the University Hospital of Messina (Prot. 71/19). Informed consent was obtained from all subjects involved in the study.

BMD was assessed by the gold-standard dual-energy X-ray absorptiometry (DXA) at the lumbar spine (L1–L4) in anteroposterior projection and at the femoral neck using a Hologic Discovery densitometer, calibrated daily, with a coefficient of variation (CV) of 0.5%.

Participants’ fracture risks were determined using the Fracture Risk Assessment tool (FRAX), a web-based algorithm (http://www.shef.ac.uk/FRAX (accessed on 21 March 2024)) that calculates the 10-year probability of major fractures (hip, clinical spine, humerus, or wrist fractures), and the 10-year probability of hip fractures. Several clinical risk factors (i.e., age, sex, weight, height, personal history of fracture, a parental history of hip fracture, current smoking, glucocorticoids, rheumatoid arthritis, secondary osteoporosis, and alcohol consumption) were consistently considered. The femoral neck BMD was not included in the calculation of the FRAX score.

Markers of bone turnover, including alkaline phosphatase (ALP) and C-terminal collagen type 1 telopeptide (CTX), in addition to serum creatinine, calcium, phosphorus, and 25(OH)D, were also measured. Creatinine, calcium, phosphorus, and ALP were determined by routine procedures, while 25(OH)D was assessed by high-performance liquid chromatography. CTX was measured using the Elecsys 2010 Immunoassay System (Roche, Basel, Switzerland) with intraassay CVs of 1.6% to 3%, and intraassay CVs of 1.3% to 4.3%.

Glomerular filtration rates (eGFRs) were calculated from data collected at baseline using the 2021 CKD-EPI equation; the variables used to estimate log GFR were log serum creatinine [modeled as a two-slope linear spline with sex-specific knots at 62 μmol/L (0.7 mg/dL) in women, and 80 μmol/L (0.9 mg/dL) in men], sex, race, and age on the natural scale.

We defined the stages of kidney function in accordance with KDIGO classification, where G1 (normal kidney function or kidney damage with normal or increased GFR) is defined by an eGFR of 90 mL/min or more, G2 (kidney damage with mild decrease in GFR) by an eGFR between 60 to 89 mL/min, G3 (moderate decrease in GFR) by an eGFR between 30 to 59 mL/min, and G4 (severe decrease in GFR) by an eGFR between 15 to 29 mL/min [16].

All participants received 60 mg of Dmab subcutaneously every 6 months and were supplemented with oral cholecalciferol (25,000 IU bimonthly). In addition, women with an estimated inadequate dietary calcium intake were provided with calcium carbonate (500 to 1000 mg, daily) to achieve the recommended daily calcium allowance.

Statistical analysis was performed with MedCalc software (version 10.2.0.0; MedCalc Software, Mariakerke, Belgium). The Kolmogorov–Smirnov test was used to assess the normal distribution of the data. Values were reported as mean ± SD or median (interquartile range). The unpaired *t*-test or the Mann–Whitney test was used for comparisons between groups; the paired *t*-test or the Wilcoxon matched rank-sum test was used to analyze data within the group as appropriate. A Chi-Square test was performed to evaluate the relationships between categorical variables. The correlation between the variables was verified through the Spearman correlation coefficient (rho) and the association between a dependent variable, and one or more explanatory variables, was evaluated by multiple regression analysis. For all tests used, a *p* value < 0.05 was indicative of statistical significance.

## 3. Results

The main clinical characteristics of the participants (*n =* 128) are listed in Table 1. The mean age of the recruited subjects was 70.3 *±* 9.4 years, and the mean Dmab treatment duration was 3.94 *±* 1.46 years. As highlighted in Table 1, participants showed a mild reduction in kidney function with mean serum creatinine of 0.81 *±* 0.27 mg/dL and eGFR of 78.2 *±* 19.3 mL/min. All the participants were divided into four groups according to stage of kidney function: G1 (*n =* 46; mean eGFR 97.34 *±* 6 mL/min), G2 (*n =* 63; mean eGFR 74.2 *±* 9 mL/min), G3 (*n =* 17; mean eGFR 47.82 *±* 7 mL/min), and G4 (*n =* 2; mean eGFR 23.5 *±* 4 mL/min). The four groups showed a similar computed 10-year risk for major osteoporotic and hip fractures, with a different distribution of considered risk factors. BMD measurements were not significantly different among groups, nor were levels of calcium, phosphorus, and bone turnover markers (i.e., ALP and CTX). Only the 25(OH)D and PTH levels were found to be different between the G1 and G2 groups. The previous treatment (before starting Dmab) for osteoporosis, including BPs and teriparatide, was also not significantly different between groups (Supplementary Table S1). A significant improvement in BMD was observed at the femur neck in G2 (vs. G1) and G3 (vs. G1 and G2).

In the stepwise multiple regression analysis—after controlling for age, BMI, and duration of Dmab treatment—the eGFR value (ß = −0.11, SE 0.04, *p* = 0.01) was found to be significantly and independently associated with changes in lumbar spine BMD over time. The same association was maintained when the eGFR categories (ß = 3.564, SE 1.29, *p* = 0.007) were considered. Moreover, after controlling for BMI and duration of Dmab treatment, age (ß = −0.7915, SE 0.37, *p* = 0.03) and eGFR (ß = −0.3257, SE 0.1567, *p* = 0.04) were found to be significantly and independently associated with BMD change in the femoral neck over time. The association was also present when the eGFR categories were considered (ß = 8.7339, SE 4.29, *p* = 0.04) (Supplementary Table S2).

## 4. Discussion

This study investigated the impact of kidney function on BMD change during Dmab treatment in osteoporotic postmenopausal women. Dmab increased BMD in all participants; in particular, the BMD increase was higher in women with lower eGFR, and particularly in groups G2 and G3. This was a remarkable result, as reduced kidney function can be considered a further risk factor for fractures in aging individuals [17].

The efficacy of Dmab had previously been observed in different settings and across a wide range of treatment durations [18], even in advanced CKD and hemodialysis patients [19,20].

Our results were consistent with those of the FREEDOM study, and support the efficacy of Dmab in postmenopausal women with mild and moderate impairment of kidney function as well [11,18]. Interestingly, the largest improvement in BMD was detected in participants with lower eGFR values. Although poorer kidney function can interfere with bone health [21,22], it did not impair Dmab action on bone tissue. All participants received adequate vitamin D supplementation and were advised to improve their dietary calcium intake. The vitamin D status is often inadequate in subjects with impaired renal function, which can lead to an alteration of calcium metabolism [23,24]. Inadequate levels of vitamin D can also exacerbate bone metabolism issues by enhancing osteoclastic bone resorption, thus increasing bone loss through the secondary rise in PTH levels [25]. Vitamin D plays a vital role in maintaining muscle mass and function, which reduces the risk of falls and the resulting fragility fractures [26]. Sufficient vitamin D levels were observed in the participants, and vitamin D supplementation prevented them from suffering further incident fractures; this adjuvated Dmab in order to maintain or improve BMD. Renal function may also predict BMD change when other anti-osteoporotic drugs are administered. Post hoc analysis of the FIT study showed that alendronate was equally effective in increasing femoral neck and spine BMD, and reducing the incidence of vertebral and non-vertebral fractures in women with and without reduced eGFR. In contrast, the increase in total hip BMD with alendronate varied according to renal function at baseline, with a slightly greater increase in women with reduced eGFR [27].

Another trial with teriparatide (rhPTH) also showed that BMD changes were equally distributed among the study groups. Compared with patients with normal renal function, patients with renal failure were older, shorter, had lower weight, were postmenopausal for a longer time, and had lower lumbar spine and femoral neck BMD values at baseline, yet teriparatide significantly increased lumbar spine and femoral neck BMD values in each renal function subgroup, and there was no evidence that these increases were impaired by renal failure [28].

A meta-analysis of nine clinical studies conducted with risedronate came to similar conclusions. The evaluation of changes in serum creatinine from baseline showed no difference in renal function between the placebo and risedronate 5 mg groups in any renal dysfunction subgroups at any time point [29].

In our study, the percentage of patients who had previously received rhPTH was equally distributed across the study groups, showing that the observed effects on BMD were due to Dmab and not through prior rhPTH treatment. Therefore, the observed results were likely attributable to the fact that high-risk patients, particularly those with the worst baseline BMD and eGFR, were the ones most likely to benefit from the initiation of Dmab therapy. Additionally, it can be speculated that renal impairment may play a role, as these patients are expected to experience higher and more frequent fluctuations in PTH levels between Dmab doses. A minimally higher PTH concentration was observed in the G2 group in association with a lower—but above-sufficiency— 25(OH)D level; the upward fluctuation of PTH could further stimulate the effect of Dmab on bone remodeling, even promoting its anabolic effect in a similar manner to that observed for intermittent rhPTH administration [30].

In addition to our findings, recent research has shown that Dmab not only improves BMD but can also promote a reno-protective effect. A study by Miyaoka et al. demonstrated that Dmab treatment significantly increased eGFR in osteoporotic patients, probably by the lowering of serum phosphorus levels. This is associated with a lower risk of CKD progression [31]. High phosphorus levels are known to stimulate fibroblast growth factor 23 (FGF23) production by osteocytes, which in turn may lead to adverse effects on both bone and cardiovascular health. Therefore, a Dmab-related reduction in serum phosphorus may also attenuate the FGF23 release, further enhancing its positive impact on bone health and kidney function [32].

Moreover, the kidney is an organ capable of metabolizing a variety of hormones and compounds. Accordingly, it may be speculated that a reduction in renal filtration can lead to a relative accumulation of these substances, some of which may contribute to the maintenance of bone tissue, such as insulin, cortisol, and testosterone. The role of insulin is crucial for bone metabolism; it has anabolic effects on bone tissue, promoting the differentiation and function of osteoblasts, which stimulate bone formation. Insulin deficiency and resistance, which are common in conditions such as diabetes, are associated with reduced BMD and an increased risk of fragility fractures [33].

Finally, we recognize that this study has some limitations, mainly represented by the retrospective design, the small sample size, the uneven number of patients in the groups identified by renal function, and the selection of only postmenopausal women by protocol. We also acknowledge all eGFR estimation formulas sometimes scatter considerably around the true value, especially if renal function is not, or is only slightly, impaired. Estimation formulas for the eGFR have only been formally evaluated for the ≤60 mL/min range. This leads us to question whether the CKD-EPI equation could differentiate between groups 1 and 2 with sufficient discriminatory power. Furthermore, the effect of increased efficacy also appears to be consistently confirmed in group 3. Some fracture risk factors (e.g., certain comorbidities or medication use other than glucocorticoids) not accounted for in the statistical analysis may have contributed to the study result, although we reiterate that the participants showed a not-significantly different 10-year fracture risk as assessed by validated algorithms. This research did not focus on fractures, but on BMD—which, nevertheless, remains the main determinant of bone strength—and investigated the role of different renal function stages on BMD changes in postmenopausal osteoporotic women treated with Dmab. These findings could help physicians adequately manage osteoporosis and fracture risk in aging populations. However, further prospective studies are needed to comprehensively investigate the association between renal function and Dmab, possibly including men and targeting fragility fractures.

## 5. Conclusions

This study highlights the sustained improvement in BMD in osteoporotic postmenopausal women receiving Dmab in real-life settings. BMD change, both at the lumbar spine and femoral neck, was closely related to renal function, and a larger BMD gain was obtained in women having lower eGFR. These findings indicate that renal function is a significant determinant of Dmab’s effectiveness in improving BMD, reinforcing the advantage of personalized treatment approaches in postmenopausal osteoporosis.

## Figures and Tables

**Table 1:** Main characteristics of participants. Data are expressed as mean ± SD. Groups are defined according to stages of kidney function (G1 > G2 > G3 > G4); BMI = body mass index; DXA = dual-energy X-ray absorptiometry; BMD = bone mineral density; PTH = parathyroid hormone; 25(OH)D = 25-hydroxyvitamin D; ALP = alkaline phosphatase; CTX = C-terminal collagen type 1 telopeptide. * *p* < 0.05 vs. G1; ^#^ *p* < 0.05 vs. G2; ^§^ *p* < 0.05 vs. G3.

	Total (n = 128)	G1 (n = 46)	G2 (n = 63)	G3 (n = 17)	G4 (n = 2)
Clinical features					
Age (years)	70.30 ± 9.3	69.3 ± 8.23	69.9 ± 9.93	74.35 ± 7.96	64 ± 7.07
Weight (kg)	60.90 ± 12.3	61.6 ± 11.5	59.8 ± 11.37	61.56 ± 13.2	63 ± 5
Height (cm)	157.0 ± 6.71	156.4 ± 7.31	157.9 ± 6.5	156 ± 5.71	152 ± 4.26
BMI (Kg/m^2^)	24.68 ± 5	25.08 ± 5.67	24 ± 4.8	25.1 ± 4.6	27.1 ± 1.95
Previous fracture [n(%)]	103 (72)	31 (64)	49 (75)	12 (83.3)	2 (100)
Parent fractured hip [n(%)]	77 (51.51)	23 (60)	35 (46)	7 (44.4)	2 (100)
Current smoking [n(%)]	32 (22.72)	9 (28.8)	16 (18)	4 (16.6)	0 (0)
Glucocorticoids [n(%)]	8 (6.06)	3 (6.6)	2 (5)	2 (11.1)	1 (50)
Rheumatoid arthritis [n(%)]	6 (4.54)	4 (4.4)	1 (3.33)	0 (0)	0 (0)
Secondary osteoporosis [n(%)]	55 (40.9)	15 (37.7)	29 (43.3)	7 (44.4)	1 (50)
Alcohol ≥ 3 units/day [n(%)]	30 (21.96)	10 (20)	12 (25)	4 (11.1)	1 (50)
10-year probability of fractures					
- Major osteoporotic (%)	29.33 ± 13.23	27.5 ± 13.94	30.04 ± 12.2	32.15 ± 14.83	37 ± 1.41
- Hip fracture (%)	14.66 ± 13	13.2 ± 12.5	14.82 ± 12.5	18.89 ± 16.6	10.2 ± 2.75
Dmab treatment duration (years)	3.94 ± 1.46	3.42 ± 2.04	4 ± 2.5	4.09 ± 2.63	4.5 ± 2
Bone mineral density					
- Lumbar spine BMD at baseline (gr/cm^2^)	0.75 ± 0.18	0.781 ± 0.2	0.729 ± 0.17	0.791 ± 0.15	0.655 ± 0.04
- Femoral neck BMD at baseline (gr/cm^2^)	0.59 ± 0.14	0.619 ± 0.13	0.577 ± 0.15	0.579 ± 0.1	0.56 ± 0.05
- Lumbar spine BMD change (%)	9.9 ± 10	8.38 ± 10.3	10.38 ± 8	8.5 ± 7.63	11.9 ± 6.96
- Femoral neck BMD change (%)	11 ± 27.6	6 ± 15.74	13.14 ± 14 *	18.9 ± 19 *^#^	11.4 ± 12.2
Laboratory data					
- eGFR CKD-EPI (mL/min)	78.2 ± 19.3	97.34 ± 6	74.2 ± 9.4 *	47.82 ± 7.86 *^#^	23.5 ± 3.5 *^#§^
- PTH (pg/mL) [reference range: 15–65]	65 ± 43	52.1 ± 33	72.6 ± 30 *	60.1 ± 39.6	31.2 ± 14.2
- 25(OH)D (ng/mL) [reference range: 30–80]	43.06 ± 15.4	46.4 ± 15.1	39.9 ± 11.1 *	40 ± 27.3	37.7 ± 2.7
- albumin corrected calcium (mg/dL) [reference range: 9–10.5]	9.29 ± 1.4	9.26 ± 0.35	9.39 ± 0.43	9.32 ± 0.48	9.12 ± 0.8
- phosphorus (mg/dL) [reference range: 3–4.5]	3.18 ± 0.34	3.11 ± 0.15	3.21 ± 0.32	3.19 ± 0.18	3.25 ± 0.11
- ALP (U/L) [reference range: 30–120]	91.15 ± 23.19	89.25 ± 19.14	92.25 ± 13.12	90.25 ± 10.2	88.2 ± 9
- CTX (ug/L) [reference range: 0.14–1.35]	0.61 ± 0.24	0.58 ± 0.21	0.63 ± 0.32	0.61 ± 0.23	0.59 ± 0.2

## Data Availability

The data presented in this study are available on request from the corresponding author.

## Supplementary Materials

The following supporting information can be downloaded at: https://www.mdpi.com/article/10.3390/jcm13206239/s1, Table S1: Frequency table of osteoporotic medical treatment before starting Dmab, Table S2: Multivariate analyses.
