# Supplementary material for: Renal Function Is Associated with Changes in Bone Mineral Density in Postmenopausal Osteoporotic Women Treated with Denosumab: Data From a Retrospective Cohort Study
Source: J Clin Med. 2024 Oct 19;13(20):6239. doi: 10.3390/jcm13206239 (PMC11514604; doi:10.3390/jcm13206239)
Supplement: Supplementary file 1 [file jcm-13-06239-s001.zip › jcm-3205589-supplementary.pdf]

Supplementary table 1: Frequency table of osteoporotic medical treatment before starting Dmab.

**Groups**	**Previous treatments**			
	**BPs**	**rhPTH**	**BPs>rhPTH**	**Naive**
**G1** (n=46)	27	3	12	4
**G2** (n=63)	37	4	16	6
**G3** (n=17)	9	2	5	1
**G4** (n=2)	0	0	1	1

BPs= bisphosphonates (alendronate, risedronate, ibandronate); rhPTH= teriparatide, Naïve= patients who did not take any osteoporotic treatment before starting Dmab. BPs were assumed for a period of 3-5 yrs. Teriparatide was administered for a course of 24 months in all the receiving patients.

Supplementary table 2: Multivariate analyses.

Model 1

Dependent Y	Lumbar Spine BMD change

Enter variable if P<	0,05
Remove variable if P>	0,1

Sample size	128
Coefficient of determination R^2	0,0703
R^2-adjusted	0,05838
Multiple correlation coefficient	0,2651
Residual standard deviation	8,5804

Regression Equation

Independent variables	Coefficient	Std. Error	t	P
(Constant)	17,8736			
eGFR CKD-EPI	-0,1171	0,04822	-2,429	0,0175

Zero order correlation coefficients

Variable	r
BMI	-0,0686
Age	0,0555
eGFR CKD-EPI	-0,265
Dmab treatment duration	0,194

Model 2

Dependent Y	Lumbar Spine BMD change

Enter variable if P<	0,05
Remove variable if P>	0,1

Sample size	128
Coefficient of determination R^2	0,08824
R^2-adjusted	0,07655
Multiple correlation coefficient	0,2970
Residual standard deviation	8,4973

Regression Equation

Independent variables	Coefficient	Std. Error	t	P
(Constant)	2,2871			
Stage of kidney function	3,5648	1,2975	2,747	0,0075

Variables not included in the model
BMI
Age
Dmab treatment duration

Zero order correlation coefficients

Variable	r
BMI	-0,0686
Age	0,0555
Stage of kidney function	0,297
Dmab treatment duration	0,194

Model 3

Dependent Y	Femoral Neck BMD change

Enter variable if P<	0,05
Remove variable if P>	0,1

Sample size	128
Coefficient of determination R^2	0,08615
R^2-adjusted	0,06302
Multiple correlation coefficient	0,2935
Residual standard deviation	27,3378

Regression Equation

Independent variables	Coefficient	Std. Error	t	P
(Constant)	90,0387			
Age	-0,7915	0,3722	-2,127	0,0366
eGFR CKD-EPI	-0,3257	0,1567	-2,078	0,0409

Variables not included in the model
BMI
Dmab treatment duration

Zero order correlation coefficients

Variable	r
BMI	-0,0761
Age	-0,190
eGFR CKD-EPI	-0,184
Dmab treatment duration	0,165

Model 4

Dependent Y	Femoral Neck BMD change

Enter variable if P<	0,05
Remove variable if P>	0,1

Sample size	128
Coefficient of determination R^2	0,08423
R^2-adjusted	0,06104
Multiple correlation coefficient	0,2902
Residual standard deviation	27,3666

Regression Equation

Independent variables	Coefficient	Std. Error	t	P
(Constant)	48,4504			
Age	-0,7832	0,3721	-2,105	0,0385
Stage of kidney function	8,7339	4,2906	2,036	0,0451

Variables not included in the model
BMI
Dmab treatment duration

Zero order correlation coefficients

Variable	r
BMI	-0,0761
Age	-0,190
Stages of kidney function	0,181
Dmab treatment duration	0,165
